# Adherence to self-administering interferon-β1a using RebiSmart® device in Mexican patients with relapsing multiple sclerosis

**DOI:** 10.1371/journal.pone.0230959

**Published:** 2020-04-20

**Authors:** Sergio Sauri-Suárez, Sandra Quiñones-Aguilar, Arturo Contreras-Marín, Erik Omar Ramiro-Guerrero, David Zúñiga-García, Leticia Salinas-Vázquez, Leonardo Llamas-López, Carolina León-Jiménez, Adriana García-Martell, Iliana González-Hernández, Erwin Chiquete, Silvia García

**Affiliations:** 1 Department of Neurology, Centro Médico Nacional “20 de Noviembre”, ISSSTE, Mexico City, Mexico; 2 Department of Neurology, Hospital Central Militar, SEDENA, Mexico City, Mexico; 3 Department of Neurology, Hospital General Naval de Alta Especialidad, SEMAR, Mexico City, Mexico; 4 Department of Neurology, Hospital Regional “Gral. Ignacio Zaragoza”, ISSSTE, Mexico City, Mexico; 5 Department of Neurology, Hospital Regional Toluca, ISSEMyM, Toluca City, Mexico; 6 Department of Neurology, Hospital Regional “Valentín Gómez Farías”, ISSSTE, Zapopan City, Mexico; 7 N&I Medical Department of Merck Biopharma, Naucalpan, Mexico; 8 Department of Neurology, Instituto Nacional de Ciencias Médicas y Nutrición Salvador Zubirán, Mexico City, Mexico; Heinrich-Heine-Universitat Dusseldorf, GERMANY

## Abstract

**Background:**

Adherence to disease-modifying therapies is determinant to attain maximal clinical benefit in multiple sclerosis (MS). RebiSmart® is an electronic auto-injector for subcutaneous delivery of interferon β-1a (INF-β1a) that monitors adherence by featuring a log of each drug administration for objective evaluation. The aim of this study was to assess long-term adherence to INF-β1a by using the RebiSmart® device in Mexican patients with relapsing MS.

**Methods:**

This is an observational multicenter study on patients with relapsing MS treated with INF-β1a subcutaneously delivered by the RebiSmart® device. Adherence was computed as the number of injections received during the study period divided by the number of injections scheduled and expressed as percent.

**Results:**

A total of 66 patients from 6 specialized MS centers were evaluated (45 females and 21 males, mean age 43.91±13.32 years). Mean adherence was 79.51±18% (median: 85.54%, range: 34.4–100%). During a median follow-up of 27.5 months (mean 33.36±29.39 months) the annualized relapse rate had a mean of 0.50±1.63. Mean initial EDSS was 1.90±1.52, and mean EDSS at the end of follow-up was 1.80±1.74. Compared with their counterparts, the mean number of relapses was significantly lower among patients with high (>80%) adherence (0.25±0.44 *vs* 0.67±92 relapses, respectively; *P* = 0.03). The proportion of relapse-free patients was 75.0% among patients with high adherence and 53.3% in low-compliant patients (*P* = 0.06). High adherence patients presented lower rates of EDSS worsening ≥1.0 at the end of treatment, as compared with low-compliant patients (11.1% *vs* 43.3%, respectively; *P* = 0.003). High schooling (>12 years) was the only predictor of a high adherence (OR: 2.97, 05% CI: 1.08–1.18; *P* = 0.03) and of being relapse-free during follow-up (OR: 3.22, 05% CI: 1.12–9.23; *P* = 0.03).

**Conclusion:**

Adherence to INF-β1a using RebiSmart® in this Mexican cohort with MS was moderate, but associated with low relapse rate and influenced by high schooling.

## Introduction

Multiple sclerosis (MS) is the most frequent chronic dysimmune disease of the central nervous system (CNS) [1]. It is characterized by entirely or partially reversible episodes of neurologic disability, usually lasting days or weeks. After a course of 10 to 20 years of an initial relapsing clinical behavior (relapsing MS), a progressive clinical pathway can develop leading to impaired mobility and cognition (secondary progressive MS) [1]. However, nearly 15% of patients have a progressive course from onset (primary progressive MS). There are approximately 400,000 cases of MS in the United States, and around 2.5 million worldwide [1,2]. The prevalence in Mexico is intermediate, estimated to be 5–20 per 100,000, for a total population affected of 6,200 to 24,800 individuals [3].

Disease-modifying therapies (DMTs) are available to decrease the frequency of relapses, to prevent neurologic disability and to limit the accumulation of focal white-matter lesions identifiable on magnetic resonance imaging (MRI). Nevertheless, there is no DMT that completely prevents or reverses the disease course [4].

Treatment adherence in conceptualized as the magnitude to which the behavior of a given person corresponds with recommendations of the healthcare provider. Especially for long-term treatments, adherence is of significant importance in achieving full beneficial effects. As a consequence, no adherence is defined as the “failing to fill prescriptions, delaying prescription fills, reducing the strength of the dose taken, and or reducing the frequency of administration. It can also include the failure to keep appointments or to follow the recommended lifestyle or dietary changes [5,6].” The availability of DMTs in low- to middle-income countries and adherence of MS patients to a medication that is not curing their disease are two critical challenges nowadays. This study aimed to evaluate the long-term adherence to interferon β-1a (INF-β1a) using the RebiSmart® device in Mexican patients with relapsing MS.

## Methods

This study was an observational, prospective and multicenter investigation about Mexican patients with relapsing MS who were treated from December 2009 to April 2018 with INF-β1a delivered subcutaneously by using the RebiSmart® electronic auto-injector device. A total of 6 centers participated with the recruitment and registering of 66 case records: *Centro Médico Nacional “20 de Noviembre”*, *ISSSTE* (n = 20), *Hospital Central Militar*, *SEDENA* (n = 20), *Hospital Regional “Gral*. *Ignacio Zaragoza”*, *ISSSTE* (n = 11), *Hospital Regional “Valentín Gómez Farías”*, *ISSSTE* (n = 7), *Hospital Regional Toluca*, *ISSEMyM* (n = 6) and *Hospital General Naval de Alta Especialidad*, *SEMAR* (n = 2). In all these centers, availability of DMTs is universal to all MS patients accepting pharmacological treatment, which contrasts with the limited availability of DMTs for the rest of the country covered by other public healthcare providers. Clinical evaluation visits were scheduled for 3±1 months at each participating center. Patients were instructed about common and expected adverse reactions to INF-β1a, and a brief interview about this topic was held in every clinical assessment visit. Telephone contact was also established to collect information on adverse reactions. Institutional Review Board and Ethics Committee *Centro Médico Nacional “20 de Noviembre”*, *ISSSTE* approved the study protocol. Signed informed consent was collected from all patients. For participants aged <18 years, signed informed consent from relatives or legal proxies was also obtained.

The selection criteria were: patients 16 years of age and older, who were affected by relapsing MS according to 2010 McDonald criteria (for patients included before 2010, retrospective analysis of compliance with 2010 McDonald criteria were required for database inclusion and analysis), who were on DMT with INF-β1a using the RebiSmart® device, and who provided electronic data of adherence stored in the device using the Mitra® software (version 1.5) from the start of treatment to the last clinical follow-up visit. Patients diagnosed with the clinically isolated syndrome (CIS), or having primary or secondary progressive MS with no relapses were excluded. Patients were invited to participate at the time of routine clinical visits for device allocation. The primary study endpoint was adherence to INF-β1a over the treatment period (i.e., from the start of INF-β1a treatment to the last revision or treatment discontinuation). Adherence was calculated as the number of injections received during the study period divided by the number of injections scheduled and multiplied by 100. Adherence was quantified by using the data (i.e., dosage, time, and date) automatically recorded by RebiSmart®. Other variables included in database were evaluated clinical and demographic characteristics, disease relapses during the study period, treatment discontinuation, reasons for treatment discontinuation and previous treatments. Annualized relapse rate (ARR) was calculated as the total number of relapses experienced in the cohort divided by the total number of days in the study, and the ratio multiplied by 365. MS relapse was defined as the reappearance of neurological symptoms during at least 24 ho after a period with stable neurological status or after at least 30 days of improvement. The Expanded Disability Status Scale (EDSS) score was estimated at the time of starting IFN β-1a treatment and at the last clinical follow-up visit.

The adherence rate to INF-β1a is expressed as the percent of valid dosages among total scheduled injections. Relative frequencies are expressed as percentages. For the relevant relative frequencies, 95% confidence intervals (CI) were calculated by the adjusted Wald method. Continuous variables with normal distribution are expressed as arithmetic means and standard deviations (SD). Non-parametric continuous variables are expressed as medians with minimum and maximum or interquartile range, as corresponded. Pearson chi-square or Fisher exact tests were used to test differences in proportions in nominal variables for bivariate analyses. To compare quantitative variables between two groups, Student *t-*test and Mann-Whitney *U* test were performed in distributions of parametric and non-parametric variables, respectively. Multivariate analyses were constructed by forward stepwise logistic regression to find independent predictors for overall adherence >80%, of the relapse-free status, and of final EDSS >2.0. A first step selection process was performed with a p set at < 0.1 in bivariate analyses. Adjusted odds ratios (OR) with the respective 95% CIs are provided. The model’s fitness was evaluated with the Hosmer-Lemeshow test for goodness-of-fit, which was considered reliable when p > 0.2. All p values were two-sided and considered as significant when p < 0.05. SPSS version 22.0 for windows (SPSS Inc., Chicago, IL.) was used in all calculations.

## Results

Among 69 patients screened, 3 patients with CIS were excluded, and a total of 66 individuals were analyzed (45 females and 21 males, mean age 43.91±13.32 years, median age: 43 years, range: 16–74 years) (Table 1). In all, 40 patients (60.6%) were enrolled in two hospitals *Centro Médico Nacional “20 de Noviembre”*, *ISSSTE* (n = 20), and *Hospital Central Militar*, *SEDENA* (n = 20). No relevant demographic and clinical differences were found in the cohort composition by the participating center.

**Table 1:** Baseline characteristics of the cohort (n = 66).

Characteristics	All patients (n = 66)	Adherence ≤80% (n = 30)	Adherence >80% (n = 36)	P value
Female sex, n (%)	45 (68.18)	21 (70.9)	24 (66.7)	0.772
Male sex, n (%)	21 (31.81)	9 (30.0)	12 (33.3)	0.772
Age, mean (SD), years	43.91 (13.32)	43.80 (12.84)	44.00 (13.88)	0.952
>12 years of school education, %	38 (57.6)	13 (43.3)	25 (69.4)	0.033
Initial EDSS, mean (SD)	1.90 (1.52)	1.88 (1.55)	1.92 (1.51)	0.930
Final EDSS, mean (SD)	1.80 (1.74)	2.03 (1.83)	1.61 (1.66)	0.330
Annualized relapse rate, mean (SD)	0.50 (1.63)	0.41 (0.83)	0.56 (2.09)	0.716
Treatment duration, mean (SD), months	33.36 (29.39)	42.03 (33.92)	26.14 (23.07)	0.030
Adherence rate, mean (SD), %	79.81 (18.01)	63.06 (13.28)	93.22 (5.58)	0.001
Treatment naïve, n (%)	35 (53.03)	19 (63.3)	16 (44.4)	0.126

EDSS denotes Expanded Disability Status Scale; SD denotes standard deviation.

**Fig 1:**
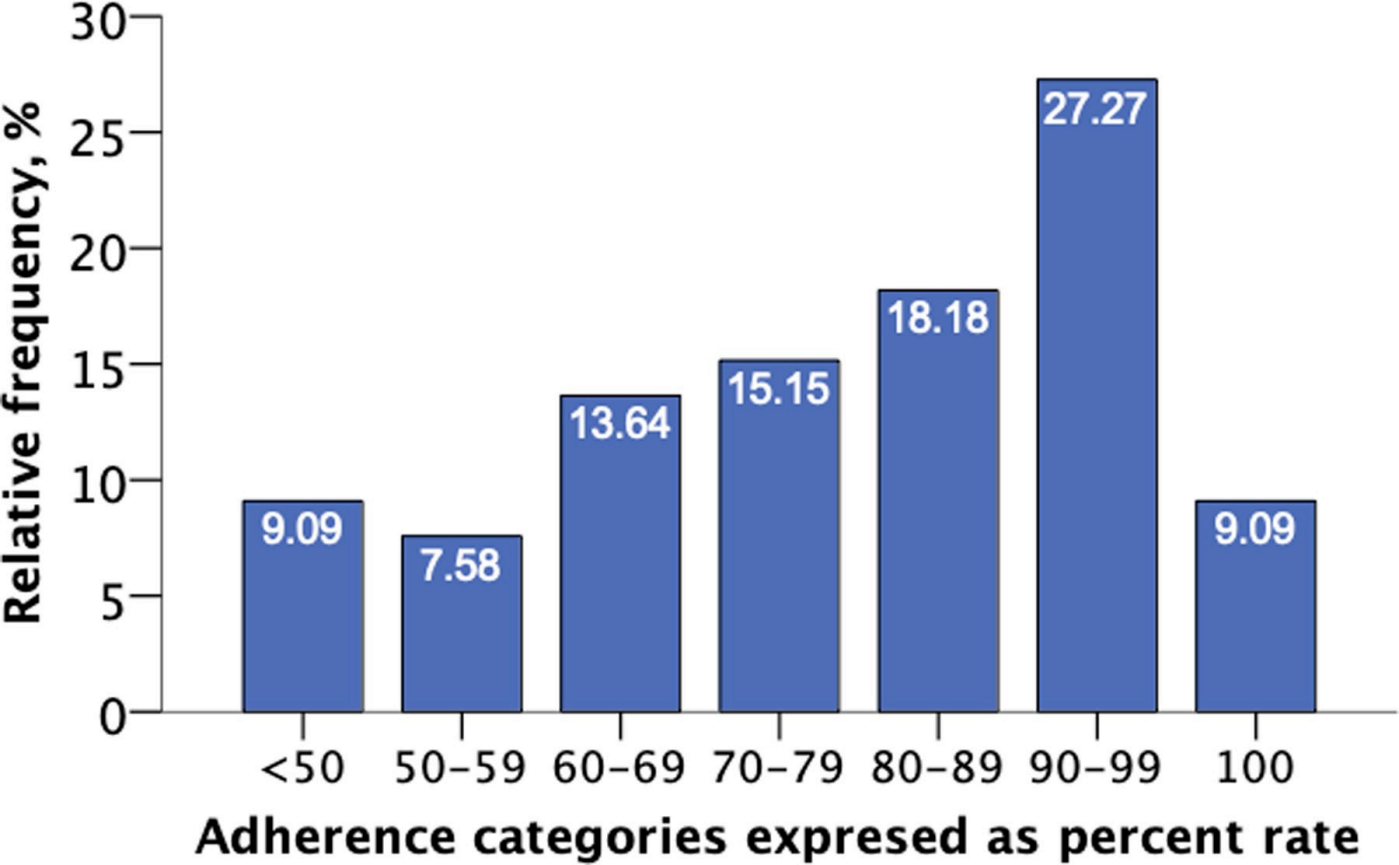
Relative frequencies of treatment adherence to interferon β-1a delivered with RebiSmart® electronic auto-injector device (n = 66).

A total of 37 patients (56.06%) were DMT-naïve. Beta interferons were the most common DMTs used in treatment-experienced patients (Table 2). Mean percent adherence was 79.51±18.0%, median: 85.54%, range: 34.4–100%, mode: 100%. In all, 54.5% of patients had overall adherence >80%, and 36.4% of patients had adherence ≥90% (Fig 1). Only 2 adverse events were reported, a case of mild elevation of liver enzymes activity and a flu-like syndrome. No clinically significant injection site reactions (ISRs) were reported, although mild and transient pain and redness were almost universally reported.

**Table 2:** Disease modifying therapies before starting interferon β-1a delivered with RebiSmart® electronic auto-injector device (n = 66).

Previous disease modifying therapy	Frequency, n (%)
No previous medication	37 (56.06)
Glatiramer acetate	9 (13.63)
Beta interferon 1a 12,000,000 IU	12 (18.18)
Beta interferon 1b 8,000,000 IU	4 (6.06)
Beta interferon 1a 22 mg	2 (3.03)
Fingolimod	2 (3.03)

**Fig 2:**
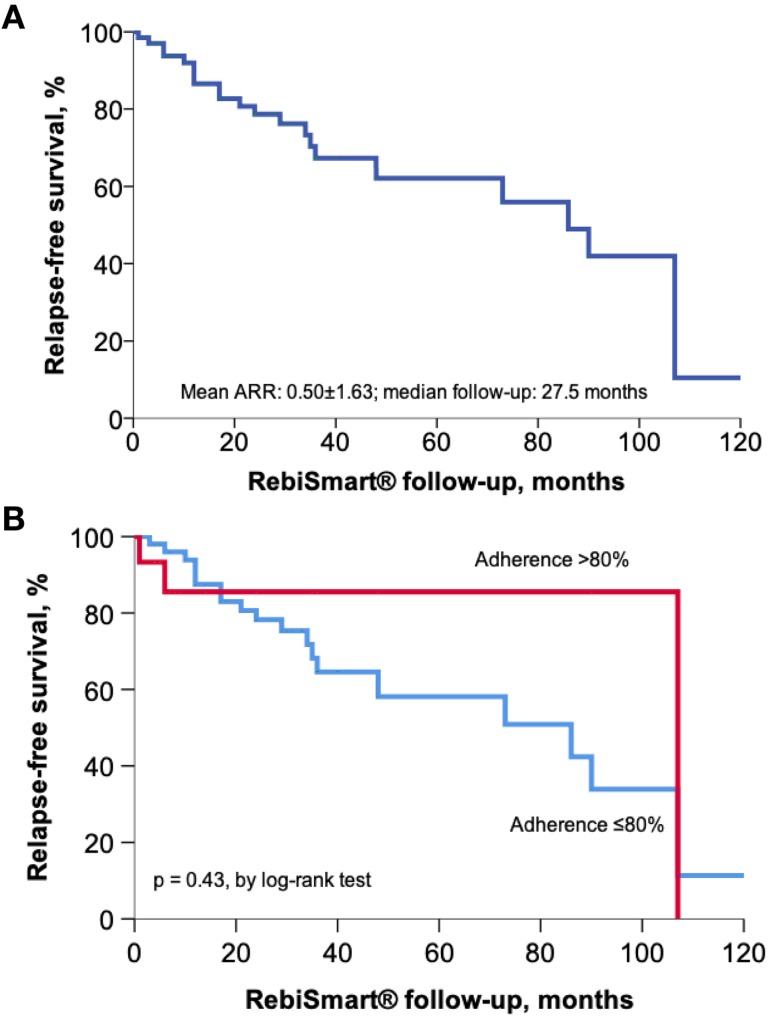
Kaplan-Meier estimates on the relapse-free probability in the total study cohort (A) and the cohort divided by treatment adherence (B). ARR denotes annualized relapse rate.

**Fig 3:**
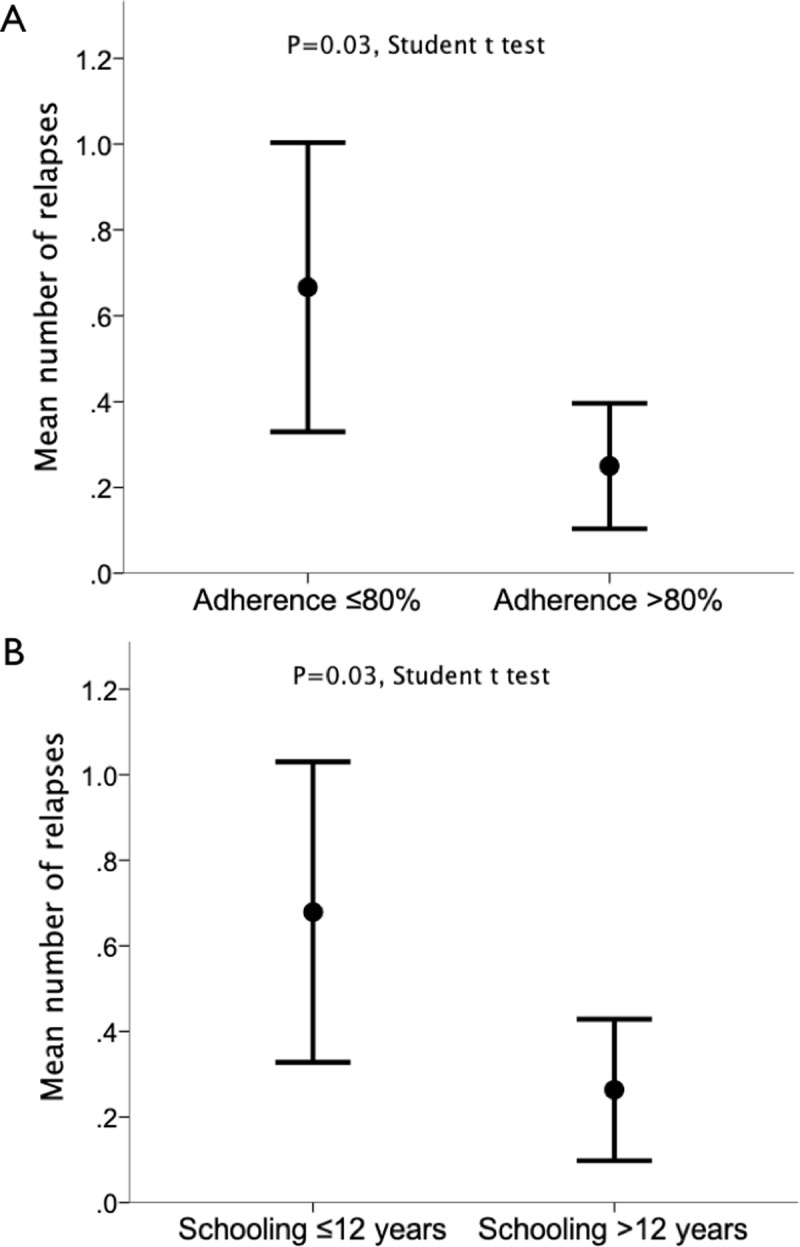
Mean number of relapses during the observation period as a function of treatment adherence (A) and years of formal school education (B). Error bars denote mean +/- 95% confidence interval of mean.

During a median follow-up of 27.5 months (mean 33.36±29.39 months), the annualized relapse rate had a mean of 0.50±1.63 (Fig 2). A total of 43 patients (65.15%) were relapse-free during the follow-up. Mean initial EDSS was 1.90±1.52, and EDSS at the end of clinical follow-up had a mean of 1.80±1.74. The mean number of relapses was significantly lower among patients with high (>80%) adherence, as compared with their counterparts with poor adherence (0.25±0.44 *vs*. 0.67±92 relapses, respectively; p = 0.03) (Fig 3A). Moreover, the mean number of relapses was significantly lower among patients with higher education (>12 years of schooling), as compared with their less educated counterparts (0.26±0.50 *vs*. 0.68±90 relapses, respectively; p = 0.03) (Fig 3B). The proportion of relapse-free patients was 75.0% among patients with high adherence, compared with 53.3% in participants exhibiting lower adherence (p = 0.06). Interestingly, patients showing high adherence presented lower rates of EDSS worsening ≥1.0 at the end of treatment, as compared with low-compliant patients (11.1% *vs*. 43.3%, respectively; p = 0.003).

In a multivariate analysis aimed to find predictors of a high adherence (i.e., >80%), the only significant independent predictor was high schooling (i.e., >12 years of formal school education) (Table 3). A high education level was also the only independent predictor of being relapse-free. In turn, after adjusting for adherence (either as a continuous or categorical variable), significant independent predictors of a final EDSS >2.0 were age >40 years, initial EDSS >1, and having at least one relapse during the observation period. The models were adjusted for age, sex, previous disease-modifying therapy, years of school education, years since multiple sclerosis diagnosis, presence of relapses, therapy adherence and EDSS at baseline.

**Table 3:** Multivariate analyses on factors associated with >80% adherence to interferon β-1a delivered with RebiSmart®, relapse-free status and expanded disability status scale >2 at the end of the observation period.*

Variables	Adjusted odds ratios (95% confidence interval)	*P* value
**Model for prediction of therapy adherence >80%**		
>12 years of school education	2.97 (1.08–1.18)	0.03
**Model for prediction of the relapse-free status**		
>12 years of school education	3.22 (1.12–9.23)	0.03
**Model for prediction of EDSS >2 at last follow-up visit**		
Age >40 years	4.91 (1.08–22.33)	0.04
Relapse during follow-up	6.80 (1.78–25.97)	0.005
EDSS >1 at baseline	7.04 (1.63–30.39)	0.009

EDSS denotes Expanded Disability Status Scale.

*Models adjusted for age, sex, previous disease modifying therapy, years of school education, years since multiple sclerosis diagnosis, presence of relapses, therapy adherence (either as continuous or categorical variable) and EDSS at baseline.

## Discussion

This study shows that adherence to INF-β1a delivered with RebiSmart® electronic auto-injector device, although moderate (>50% patients exhibited >80% adherence), results in a lower probability of relapse and it is mostly influenced by high school education. Nonetheless, the adherence rate observed in our study is higher than that found in other studies. Formal education was also associated with a low number of relapses and with a high rate of a relapse-free status. Because there is not curative treatment available nowadays, adherence to long-term DMTs aimed to control MS activity is still challenging. The most frequent causes for discontinuation of medications are disease progression, patient-perceived lack of efficacy, injection anxiety, and adverse effects including injection-site reactions, flu-like symptoms, reversible cognitive impairment, and fatigue [6–9]. Also, financial aspects, such as cost of therapies, co-payments to medical insurers, and other societal factors, are essential for the decision to leave medications, especially in low- to middle-income countries. In Mexico, some agencies of the public health system have programs for the care of MS patients, but they are not universally accessible.

According to data from the European Multiple Sclerosis Therapy Consensus Group, patients usually discontinue treatment after three years [6]. Adherence rates to DMTs range from 41% to 88%; in retrospective studies, weighted mean adherence is 53.1% versus prospective studies with weighted mean adherence 72.8%. However, data about Latin-American populations are still lacking.

Adherence to injectable DMTs and other interventional strategies is usually considered inferior in MS patients [10–16]; a retrospective study of MS patients initiating therapy with 1 of 4 injectable DMTs found that after 18 months, 11.2% of patients switched their medication, and 33.9% discontinued their treatment [17]. An adherence study on ambulatory patients carried out in Spain showed that after 2 years of starting DMT, INF-β was discontinued in 9.9% of patients, after 5 years discontinuation rose to 41.2% and after 8 years discontinuation rate was 58.7% [18]. Another study found that adherence to INF-β therapy is difficult for patients with discontinuation rates ranging from <20% to 50% within the first two years, if there is no evidence of relapse and or progression [19].

Electronic auto-injection device (RebiSmart®; Merck Serono S.A.–Geneva, Switzerland, a branch of Merck Serono S.A., Coinsins, Switzerland, an affiliate of Merck KGaA, Darmstadt, Germany) was developed for the subcutaneous administration of IFN-β1a [10–14,20]. The device simplifies the injection process and help patients to overcome injection-related issues, thereby possibly increasing adherence to treatment. Pozzilli et al. [21] in a cohort study concluded that the use of this mechanical auto-injector was a robust predictor of adherence to MS medication in a 2-year follow-up period. Adherence rates in the present study are comparable to data from D’Arcy et al. [20] and Pozilli et al. [21] studies, who evaluated adherence with the same device in MS patients. On the contrary, adherence was low in studies where the device was not used [17–19]. The RebiSmart® device offers convenience characteristics such as the multi-dose cartridge which holds one week drug dosage, an easy-to-understand mechanism, step-by-step instructions, an injection log to inform patients and the medical professionals of injection history, as well as adjustable comfort settings, which allow patients to tailor injections to improve their comfort. Needle and injection speed, time, and depth of injection can all be changed by patients who experience discomfort upon injection or who are dissatisfied with the injection process [21].

Interestingly, our patients reported only two minor adverse events, and there were no clinically significant ISRs, although flu-like symptoms were frequent. Mikol et al. [22] found that injection devices have reduced ISRs; the results in our study confirm this finding. These devices have diminished ISRs because of an ensuring correct injection technique, and therefore, ISRs are more related to the nature of DMT than with the injection technique. Mechanical auto-injector devices for subcutaneous delivery of drugs has shown to significantly improve injection tolerability compared with manual injection, in particular among patients with reduced manual dexterity or visual or cognitive impairment. The use of a mechanical auto-injector was a strong predictor of adherence to MS medication in a 2-year, observational study [23]. Surveys about patients’ satisfaction with auto-injector reported the ability to carry out the injection simply and quickly in only a few steps, with customizable technique characteristics are associated with better tolerance, and high-level adherence and user satisfaction [17,24,25].

The assessment of efficacy was not an objective of this study; however, we observed a high proportion of patients being relapse-free and with EDSS scoring that, in average, showed no worsening as compared with baseline. Adherence was associated with less probability of EDSS worsening, but it was not independently associated with a low ARR or the relapse-free status, being schooling the concealed variable that by associating with adherence is also associated with therapy outcomes.

There are some limitations of this study that need to be acknowledged for the correct interpretation of our findings. The small sample size of this study may not be powered to detect small but relevant differences and interactions, especially concerning to variables that influence adherence. Moreover, although we found that disease progression was better among patients with high (i.e., >80%) treatment adherence, no comparisons could be made with a group of patients receiving INF-β1a without the RebiSmart® device. Therefore, no size effect can be calculated on the absolute adherence to DMT by using the electronic device compared with no device usage. Also, a more extended observation period could be useful to describe very long-term adherence and non-baseline variables that may influence compliance to therapy, such as disease progression, adverse effects, aging, and availability of competing DMTs, among other factors.

## Conclusion

In conclusion, adherence to INF-β1a by using the RebiSmart® device in this Mexican cohort with relapsing MS was moderate, although comparable with studies of similar characteristics. Adherence is associated with a low number of relapses, low rates of EDSS worsening, and high schooling. Also, high schooling (>12 years) was the only predictor of a high adherence rate and of being relapse-free. In contrast, predictors of EDSS >2 at the end of follow-up were age >40 years, experiencing a relapse during the study period and EDSS >1 at baseline. Better-powered studies are necessary to demonstrate that high adherence rates are associated with treatment success in the Mexican population with MS.

## Supporting information

S1 Data (SAV)
